# Integrating machine learning and structural analysis to decipher benzo[a]pyrene-induced bladder cancer networks

**DOI:** 10.1007/s12672-026-05207-6

**Published:** 2026-05-21

**Authors:** Xinzhao Zhao, Ruize Qin, Chengquan Shen, Ding Hu, Cheng Li, Changxue Liu, Huaixi Ge, Yonghua Wang

**Affiliations:** Department of Urology, The Affiliated Hospital of Qingdao University, Qingdao, Shandong China

**Keywords:** Benzo[a]pyrene, Bladder cancer, Machine learning, Molecular docking, Molecular dynamics simulations

## Abstract

Benzo[a]pyrene (BaP), a polycyclic aromatic hydrocarbon from tobacco smoke, exhaust, and pollutants, is linked to bladder cancer (BLCA). We systematically analyzed GEO datasets to identify BaP-related differentially expressed genes (DEBRGs). By integrating network toxicology, machine learning, molecular docking, molecular dynamics, and single-cell transcriptomics, we identified 19 significant genes, among which 7 key DEBRGs were prioritized (GSK3B, SKP2, AURKB, EPHB4, KIT, NR3C2, and CA2) in BaP-mediated BLCA. SHAP analysis highlighted GSK3B and SKP2 as important genes contributing to the predictive model. Single-cell data revealed their cell-type specific expression in the tumor microenvironment. Molecular simulations detailed interactions between BaP and target proteins. This study identified critical genes in BaP-induced bladder carcinogenesis, offering insights into underlying molecular mechanisms and potential therapeutic targets.

**Supplementary Information:**

The online version contains supplementary material available at 10.1007/s12672-026-05207-6.

## Introduction

Benzo[a]pyrene (BaP) is a common polycyclic aromatic hydrocarbon (PAH) that is harmful to the environment. The formation of this substance occurs primarily due to the incomplete burning of organic substances (including tobacco, burnt food and industrial waste) [1, 2]. BaP is a high carcinogenic agent and may lead to several types of cancer such as lung cancer, colorectal cancer and hepatotoxicity [3–5]. Bladder cancer (BLCA) is a urinary tract tumor, which is often strongly unfavorable to prognosis with a high probability of recurrence at advanced stages [6, 7]. Occupational and environmental exposure to PAHs over a long period of time is regarded as an important risk factor of bladder cancer (BLCA) [8]. It is notable that long-term exposure to BaP also has a significant epidemiological association with bladder cancer [9, 10]. This implies that the molecular processes through which BaP induces bladder cancer warrant careful investigation.

Cytochrome P450 enzymes metabolize BaP into reactive forms that can bind directly to DNA and form adducts. These changes to DNA can sometimes cause mutations in genes that typically control cell development. These modifications can make the genome unstable and cell proliferation uncontrolled [11]. In addition to these direct impacts on DNA, BaP may also change how cells work, like cell cycle, programmed cell death, and how cells respond to oxidative stress [12, 13]. All of these changes can lead to the growth of tumors. Even with this information, the exact genes and signaling networks that BaP affects in bladder cancer are still mostly unknown.

The recent progress in bioinformatics, systems biology, and computational modeling provides strong methods of systematic prediction of possible chemical-protein interactions as well as the key molecular drivers of disease. Combining transcriptomic analysis and machine learning will allow the determination of cancer-related genes and their interaction with environmental carcinogens like BaP. Moreover, the structural information about the binding affinity and stability of small molecules to their target proteins can be obtained by molecular docking and molecular dynamics simulations that may provide mechanistic information on the interaction between carcinogen and protein at the atomic level. These integrative strategies assist in identifying the functional roles of BaP in regulating bladder cancer pathways and determining possible biomarkers or treatment targets.

This paper was an attempt to systematically explore the molecular pathways through which BaP promotes bladder carcinogenesis. We combined transcriptomic information, machine learning, molecular docking, and molecular dynamics simulations in order to find possible targets of BaP and evaluate their functional relevance in BLCA. Our study can identify key genes and signaling pathways regulated by BaP as an insight into its mechanism of action in tumor formation and progression. The findings not only expand our knowledge regarding the causes of bladder cancer due to BaP but also serve potential assessment of risk, early diagnosis, and personalized treatment intervention choices in BLCA.

## Materials and methods

### Data collection

**Fig. 1:**
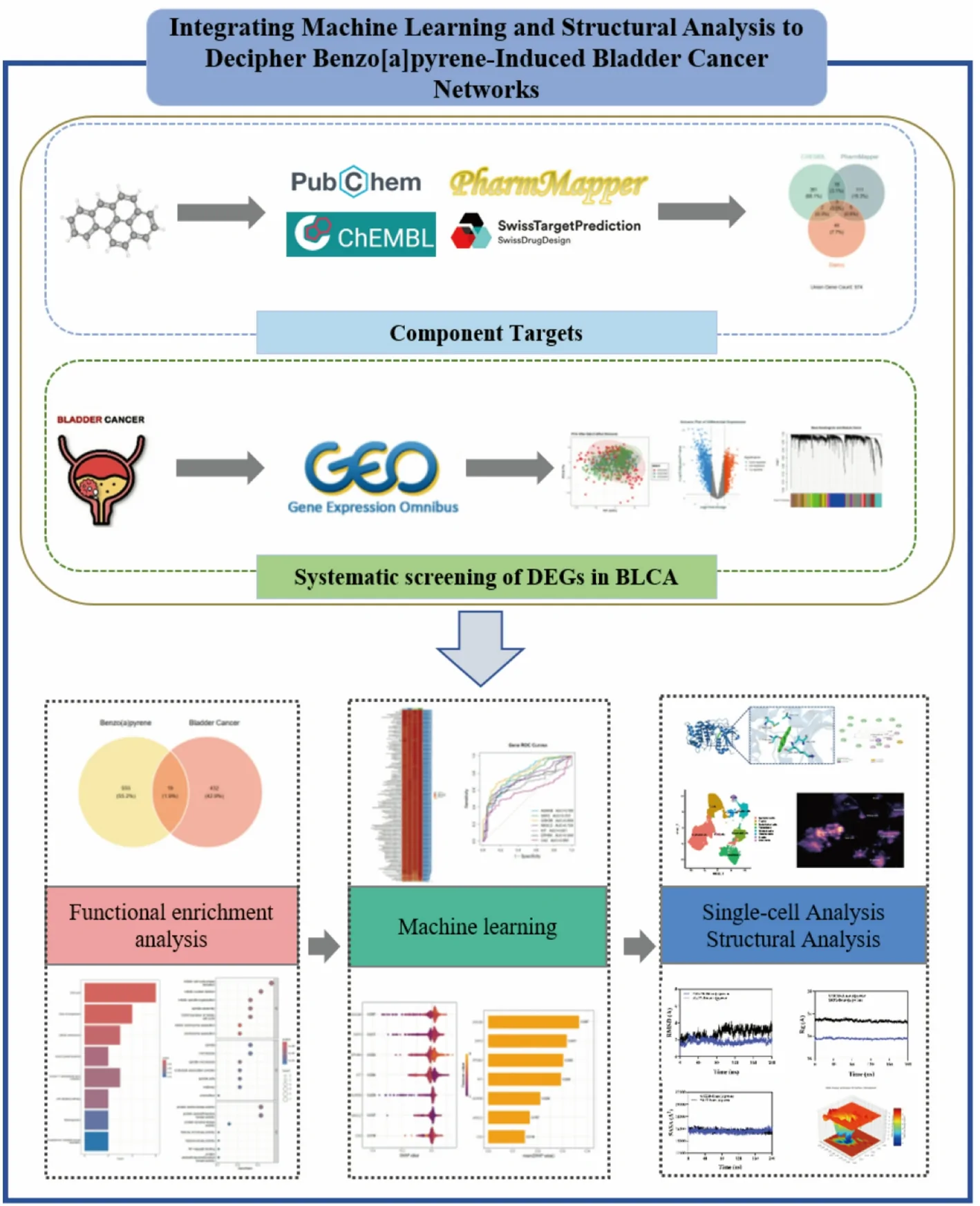
Flow-chart of dataset analysis in this paper

Raw gene expression data of the six bladder cancer datasets (GSE13507, GSE31684, GSE32894, GSE7476, GSE37815, and GSE65635) and the single-cell bladder cancer dataset (GSE222315) were downloaded from the GEO database and subsequently preprocessed. The training cohort was represented by GSE13507, GSE31684, and GSE32894, whereas the validation cohort consisted of GSE7476, GSE37815, and GSE65635. A common gene set was used to combine the expression matrices of individual datasets with the samples labeled according to their batch of origin. Surrogate Variable Analysis (SVA) was used to adjust latent confounding variables, and the ComBat empirical Bayes framework was used to correct residual batch effects. Principal component analysis (PCA) confirmed the integration as it demonstrated better intermixing of samples belonging to different batches in the corrected data, which proved that the technical variance specific to a given batch had been eliminated. The workflow diagram of the study is shown in Fig. 1.

### Acquisition of chemical components and targets of BaP

BaP was defined by the combination of several databases. Its physicochemical and biological parameters were methodically retrieved from PubMed, while canonical 2D structural descriptors (SMILES: C1 = CC=C2C3 = C4C(= CC2 = C1)C=CC5 = C4C(= CC=C5)C=C3) were obtained from the PubChem database. Toxicity profiling of BaP was conducted utilizing the ADMETlab 3.0 (https://admetmesh.scbdd.com/) and ProTox-III (https://tox.charite.de/protox3/), with the objective of predicting carcinogenicity, mutagenicity, and additional organ-specific toxicological endpoints. A tripartite method was employed in target prediction: (i) ligand-receptor interaction profiling using the ChEMBL Database (https://www.ebi.ac.uk/chembl/); (ii) chemical genomics-based predictions using SwissTargetPrediction (http://www.swisstargetprediction.ch); and (iii) 3D pharmacophore matching via PharmMapper (http://lilab-ecust.cn/pharmmapper). All predicted targets were limited to the Homo sapiens proteome.

### Differential gene expression analysis

Differentially expressed genes (DEGs) were identified through the analysis of transcriptome data using the limma package, employing FDR-adjusted P-value < 0.05 and log2FC > 0.585 (corresponding to a 1.5-fold change). The findings were plotted using ggplot2.

### Weighted gene co-expression network analysis (WGCNA)

The scale-free co-expression networks were built using the WGCNA software. Outliers were removed through hierarchical clustering based on sample quality. To achieve a scale-free topology, an appropriate soft-thresholding power was selected such that the coefficient of determination (R²) exceeded 0.9. Hierarchical clustering based on the topological overlap matrix (TOM), followed by dynamic tree cutting, was used to identify gene co-expression modules, which were subsequently merged based on module similarity. Pearson correlation was used to determine module-trait associations, where correlations > |0.5| and *P* < 0.05 were significant. Module membership (kME) value > 0.8 defined hub genes in important modules.

### Identification of BaP-associated disease targets

The hub genes identified through the intersection of DEGs in bladder cancer and WGCNA modules, along with the potential target genes of BaP, were overlapped and visualized using a Venn diagram.

### Functional enrichment analysis

The clusterProfiler package (*P* < 0.05) was used to perform GO (biological process, cellular component, molecular function) and KEGG pathway enrichment analyses to determine the most important biological processes and molecular pathways that may be related to BaP in bladder cancer, explaining its possible mechanisms in BLCA pathogenesis.

### Machine learning-based core gene screening

In order to study the role of BaP in the pathogenesis of bladder cancer, we created a complete machine learning framework to build diagnostic models using gene expression data. We created an ensemble of 127 alternative prediction models by using 12 basic algorithms: Lasso, Ridge, ENet, Stepglm, SVM, glmBoost, plsRglm, RF, GBM, XGBoost, LDA, and Naive Bayes, as well as numerous combinations of these techniques. Before model training, the data were normalized and minimally perturbed to reduce noise. We adopted a two-stage feature selection strategy. For each algorithm with embedded variable selection capability, the model was first run on the training set to automatically select significant variables, with a minimum variable threshold of 2. If fewer variables were selected, additional features were supplemented based on t-test P-values. Hyperparameters for each algorithm were optimized using 10-fold cross-validation to reduce the risk of overfitting. Model performance was assessed using internal ten-fold cross-validation, with predictive accuracy evaluated by the area under the receiver operating characteristic curve (AUC). To identify genes involved in BaP exposure, models that performed better (AUC > 0.9) underwent additional analysis. Then, the expression patterns and model-specific importance of these genes were visualized using the pheatmap package.

### SHAP for model Interpretation

To quantify the contribution of each feature to the prediction outcomes, we performed SHapley Additive exPlanations (SHAP) analysis. The SHAP values were computed using R package SHAP, which provides a standard, cooperative game-theory-based metric for feature importance. For each retained model, feature-level contributions to prediction outcomes were estimated for individual samples and integrated across samples to determine the genes with the greatest influence on model classification. This study was undertaken to ensure the interpretability of the ensemble models and to accurately identify critical gene features associated with the role of BaP in the pathogenesis of bladder cancer.

### Single-cell transcriptomic analysis

Single-cell RNA sequencing data of bladder cancer samples (GSE222315) were obtained from the GEO database and processed using the Seurat R package. After quality control, normalization, and dimensionality reduction via Uniform Manifold Approximation and Projection (UMAP), cells were clustered into distinct populations. Cell types were annotated based on canonical marker genes, and the expression patterns of differentially expressed genes were assessed across cell types. Average expression levels and the proportion of positive cells were calculated to evaluate cell type–specific expression.

### Molecular docking analysis

To investigate the potential connections between BaP and key candidate genes, molecular docking studies were conducted. The three-dimensional configuration of the target proteins were retrieved from the RCSB Protein Data Bank (RCSB PDB), while the chemical structure of BaP was obtained from the PubChem database (Table 1). Protein structures were prepared using PyMOL by removing water molecules and heteroatoms and adding hydrogen atoms. AutoDockTools were used to define the binding pocket. Before docking, the small molecule was minimized in energy at physiological pH (7.4) with the MMFF94 force field in AutoDock where rotatable bonds were assigned. AutoDock Vina was then used to perform molecular docking simulations. The binding poses and interaction patterns that emerged were visualized and analyzed using PyMOL and Discovery Studio.

**Table 1:** Target protein and PDB ID

Target protein	PDB ID
AURKB	4AF3
CA2	1CIL
EPHB4	2BBA
GSK3B	4J1R
KIT	4HVS
NR3C2	3VHV
SKP2	1FQV

### Molecular dynamics simulation

Molecular dynamics simulations were carried out with GROMACS 2022. The ligand parameters were generated using sobtop_1.0 (dev3.1) on the basis of the GAFF2 force field, with partial atomic charges being assigned by RESP method and the protein was described in terms of the AMBER14SB force field. We utilized the TIP3P water model to solvate the system in a cubic box with a minimum distance of 1.0 nm between the solute and the border of the box. To neutralize the system and get the physiological ionic strength of 0.15 M NaCl, we injected Na⁺ and Cl⁻ ions. The Particle Mesh Ewald (PME) approach was employed for long-range electrostatic interactions with a cutoff of 1.0 nm, whereas bond restrictions were managed using the LINCS algorithm. Minimization of energy was done through steepest descent steps of 3000 followed by conjugate gradient minimization steps of 2000. Simulations of production were run at NPT ensemble with a temperature of 310 K and pressure of 1 bar with a NosHoover thermostat and Parrinello-Rahman barostat with a time step of 2 fs. Standard GROMACS analysis tools were used to perform trajectory analyses such as Root Mean Square Deviation (RMSD), Root Mean Square Fluctuation (RMSF), radius of gyration (Rg), and solvent-accessible surface area (SASA) to determine structural stability and conformational dynamics. Additionally, the binding free energies between BaP and the target proteins were calculated using the Molecular Mechanics Poisson-Boltzmann Surface Area (MM-PBSA) method based on the equilibrated trajectories.

## Results

### Toxicity prediction for BaP

**Fig. 2:**
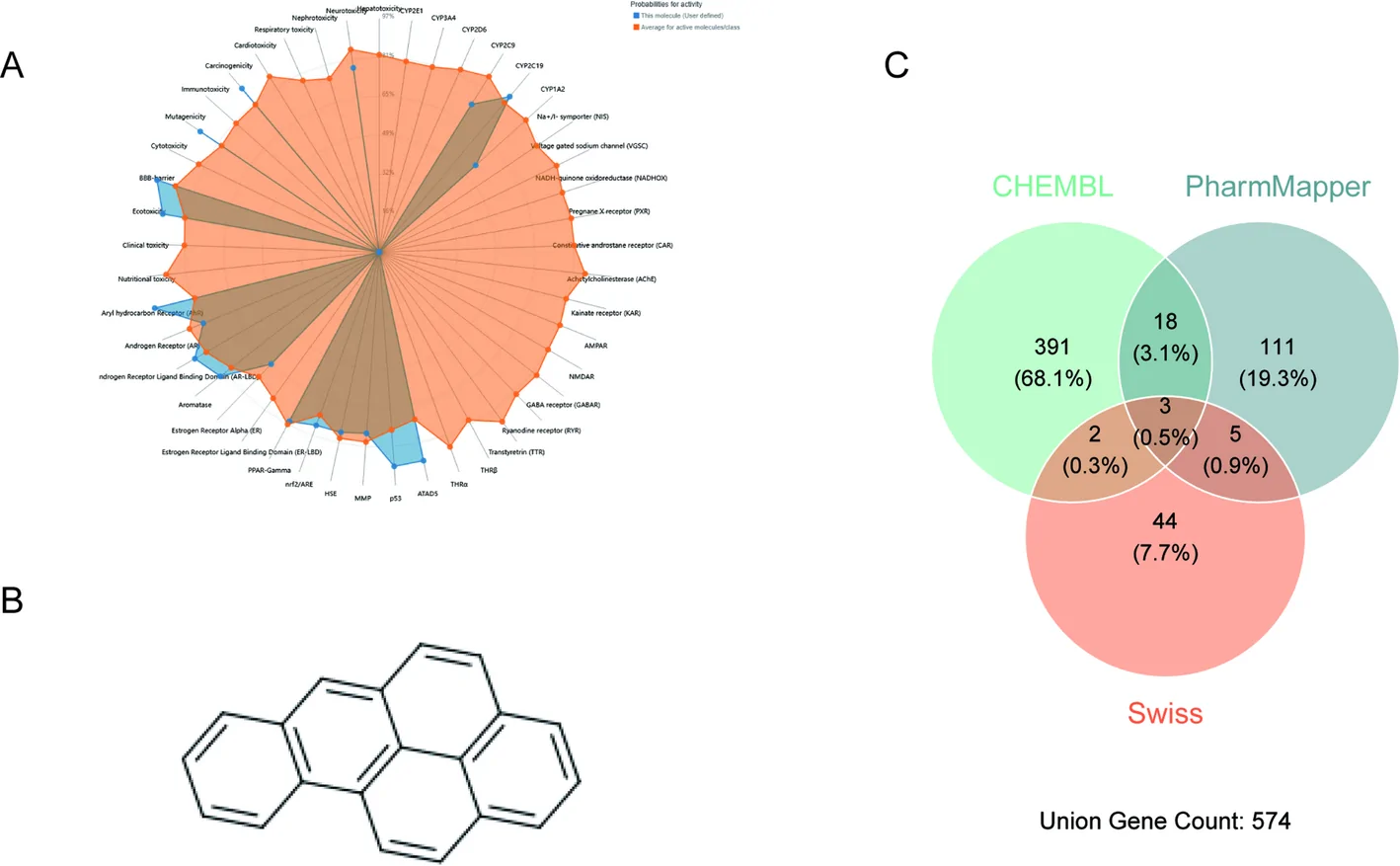
Comprehensive in silico prediction of Benzoapyrene (BaP)-associated protein targets. **A** Toxicity distribution in BaP. **B** Chemical structure of BaP. **C** Prediction of targets using ChEMBL, PharmMapper, and SwissTargetPrediction

According to the integrated toxicity prediction of ADMETlab 3.0 and ProTox-III, BaP has a significant toxicological risk. The ADMETlab 3.0 predicted high chances of carcinogenicity, mutagenicity, neurotoxicity, and respiratory toxicity, whereas the ProTox-III consistently categorized BaP as a high-confidence carcinogen and mutagen. These findings taken together suggest that BaP is highly genotoxic and multi-system toxic in nature, which can be used as toxicological evidence of its role as an environmental carcinogen that may be implicated in the initiation and progression of bladder cancer (Fig. 2A).

### Identification of BaP target proteins

Molecular structure of BaP was obtained on PubChem (Fig. 2B). In order to fully determine its possible biological targets, three complementary in silico tools were used: ChEMBL (curated bioactivity data), PharmMapper (reverse pharmacophore mapping), and SwissTargetPrediction (ligand-based similarity screening). After combining the results and eliminating duplicates, a non-redundant list of 574 unique potential targets was generated for further analysis (Fig. 2C).

### Screening of BLCA-related genes

**Fig. 3:**
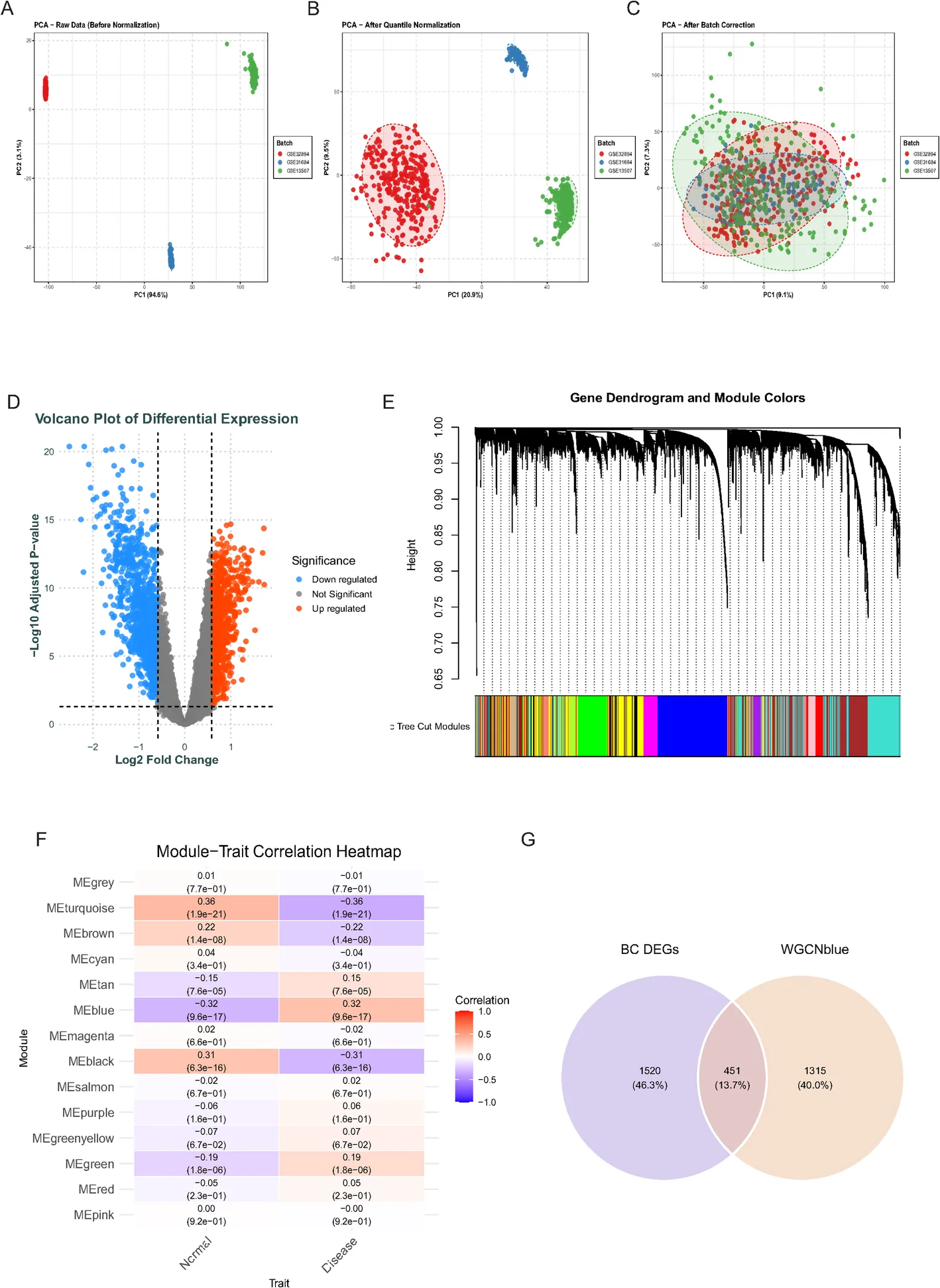
Identification of BLCA-Related Target Genes. **A**–**C** The PCA scatter plot batch correction illustrates the merging of the GSE32894, GSE31684 and GSE13507 datasets. **D** The volcano plot displays DEGs between tumor and normal samples according to logFC and significance, with red dots indicating upregulation, green dots indicating downregulation, and grey dots representing non-significant changes. **E** The WGCNA gene dendrogram illustrates hierarchical clustering based on co-expression, with the lower panel’s module colors indicating various gene modules. **F** Module-trait relationship heatmap displaying correlations between WGCNA-identified modules and sample traits; each cell shows the correlation coefficient and corresponding p-value, with red/blue indicating positive/negative correlations. **G** The Venn diagram illustrates DEGs and WGCNA blue modules, showing the overlap of genes identified by both approaches

To correct batch effects, the GSE32894, GSE31684, and GSE13507 datasets were harmonized and subjected to batch correction. PCA showed better data distribution and increased consistency (Fig. 3A-C). The corrected data were analyzed using differential expression analysis and found that 1,971 genes had significant changes in BLCA, which were visualized using a volcano plot (Fig. 3D). To establish a scale-free network architecture, the ideal soft-thresholding power (β) was calculated to perform WGCNA. The evaluation included powers from 1 to 20, with β = 4 being chosen as the lowest value that met the scale-free criterion (R² > 0.9). We identified 14 distinct co-expression modules, each represented by a unique color (Fig. 3E). Subsequent module–trait correlation analysis revealed significant associations between several modules and BLCA (*P* < 0.05) (Fig. 3F). By intersecting differentially expressed genes with WGCNA-derived module genes and eliminating redundancies, we obtained a final set of 451 BLCA-related genes for downstream analyses (Fig. 3G).

### Identification of BaP-associated BLCA targets

**Fig. 4:**
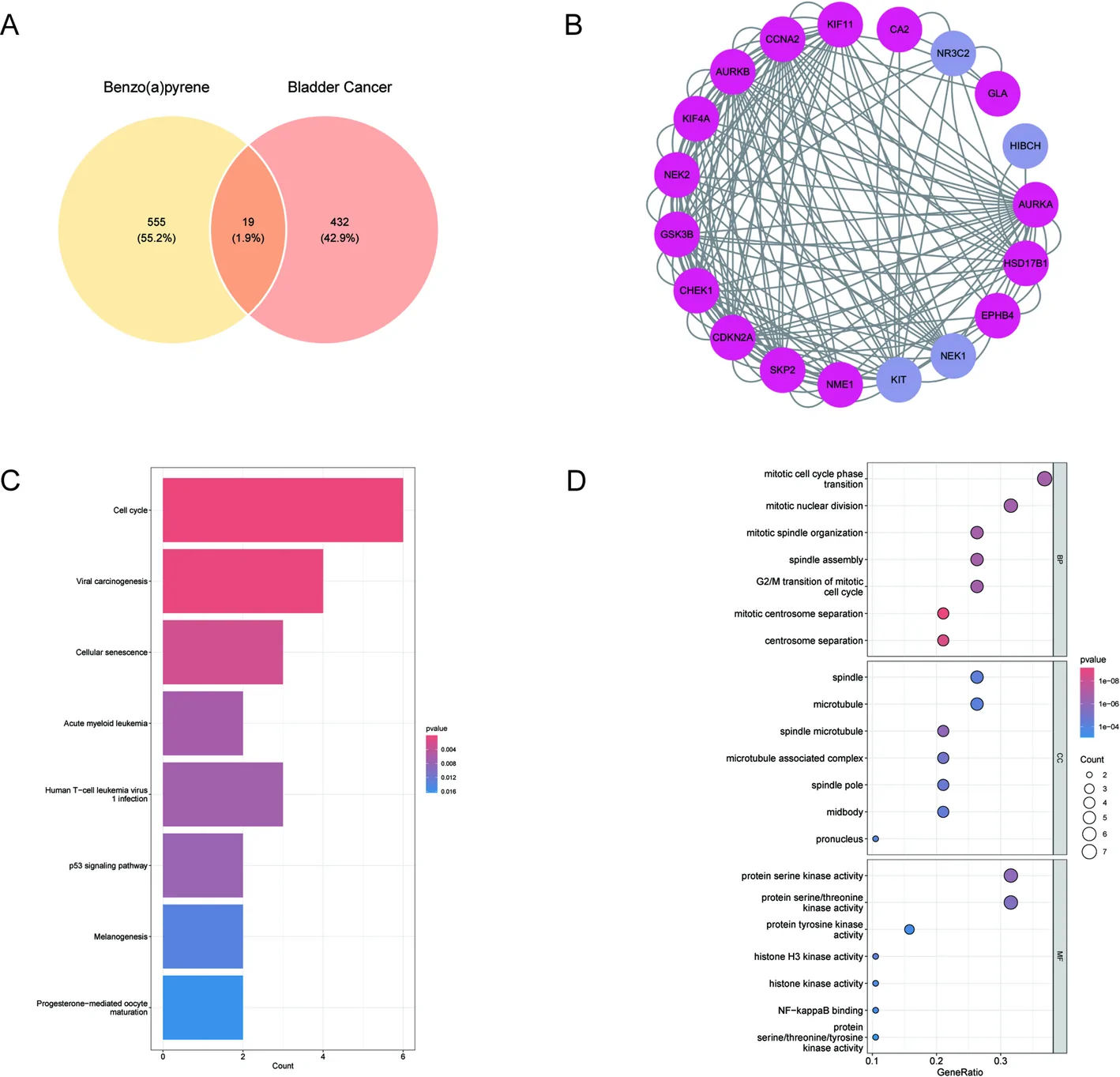
Identification of Bap-related Targets in BLCA. **A** The Venn diagram shows 19 genes that are associated with Bap exposure and BLCA (1.9%). **B** PPI network visualizing predicted protein–protein interactions among the 19 overlapping genes. **C** KEGG analysis shows enriched pathways for overlapping genes with bar graphs as well as lollipop plots. **D** GO enrichment annotates overlapping genes in BP (Biological Process), CC (Cellular Component), and MF (Molecular Function)

The intersection of BaP putative targets with BLCA-related genes identified 19 key targets, which might be involved in the induction of bladder cancer by BaP (Fig. 4A, B). Further KEGG enrichment analysis indicated that these genes were enriched in a variety of tumor-related signaling pathways, with the cell cycle being the most significant pathway, followed by p53 signaling pathway, viral carcinogenesis, and cellular senescence, implying that BaP could contribute to bladder cancer development by modulating cell proliferation, apoptosis, and virus-mediated carcinogenic processes (Fig. 4C). GO functional enrichment showed that these 19 genes were mainly related to mitosis-related processes, such as mitotic cell cycle phase transition, mitotic nuclear division, and mitotic spindle organization, suggesting that BaP may influence bladder cancer progression through dysregulated tumor cell proliferation and division. Cellular component analysis showed that these genes were enriched in spindle- and microtubule-related structures, including spindle, spindle pole, and spindle microtubules, highlighting the significant role in the assembly and function of the cell division machinery. Molecular function analysis revealed significant enrichment in kinase activities, including protein serine/threonine and tyrosine kinase activities, histone H3 kinase activity, and NF-κB binding, suggesting that BaP may regulate bladder cancer-related signaling pathways through effects on protein phosphorylation, chromatin modification, and transcription factor activity (Fig. 4D).

### Identification of hub genes in BaP-mediated bladder carcinogenesis based on machine learning

**Fig. 5:**
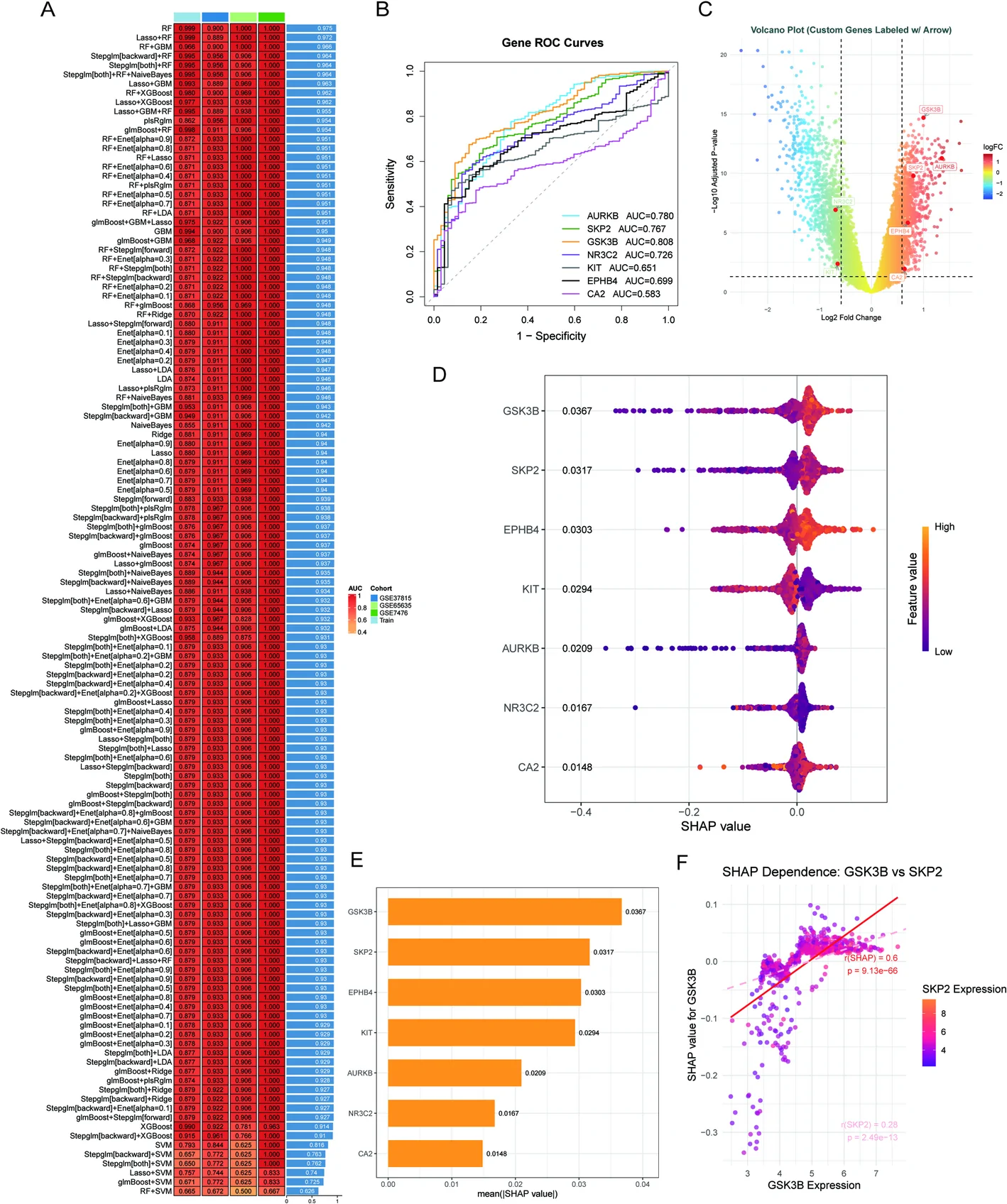
Identification of Core Genes in BaP-Induced BLCA. **A** The heatmap displays AUC values for different models across various cohorts, with models listed in the left column and AUC in the right. Cohort sources are represented by colors. **B** ROC curves illustrate model performance for key genes, with the X-axis showing the false positive rate and the Y-axis indicating sensitivity. **C** Volcano plot displays DEGs with key genes marked. **D** Violin plots display the distribution of gene expression across different conditions, with the width indicating data density and colors representing gene expression. **E** A bar chart displays the ranking of top genes based on feature importance, with longer bars signifying a higher contribution to the model. **F** SHAP dependency plot: The x-axis represents the expression of GSK3B, the y-axis represents the predictive contribution to the model, and the color of the dots indicates the expression of the core genes. The red line and the pink line respectively represent the correlation between the SHAP value of GSK3B and the expressions of GSK3B and SKP2

We conducted machine learning analysis of 19 potential targets and developed 127 predictive models to determine core genes related to BLCA associated with BaP (Fig. 5A). The random forest (RF) model was the best performer in terms of overall performance with an AUC of 0.975, which resulted in identifying 7 differentially expressed BaP-related genes (DEBRGs): AURKB, SKP2, GSK3B, NR3C2, KIT, EPHB4, and CA2 (Figure S1 A-C). ROC curves were used to further assess the diagnostic value of each gene, indicating significant differences in AUC values. GSK3B showed the highest diagnostic performance (AUC = 0.808), followed by AURKB (AUC = 0.780) and SKP2 (AUC = 0.767), all of which were highly expressed in bladder cancer patients (Fig. 5B, C). SHAP analysis was then carried out to explain the contribution of DEBRGs to model predictions and their possible mechanisms. GSK3B and SKP2 turned out to be the most influential genes, always ranked highest in multiple analyses (Fig. 5D, E). Notably, SHAP values of GSK3B increased with its expression level, showing a significant positive contribution to model predictions; similarly, SKP2 expression positively correlated with GSK3B’s SHAP values, suggesting a synergistic effect between GSK3B and SKP2 in disease prediction (Fig. 5F).

### Single-cell transcriptomic profiling reveals cell type–specific distribution of DEBRGs in bladder cancer

**Fig. 6:**
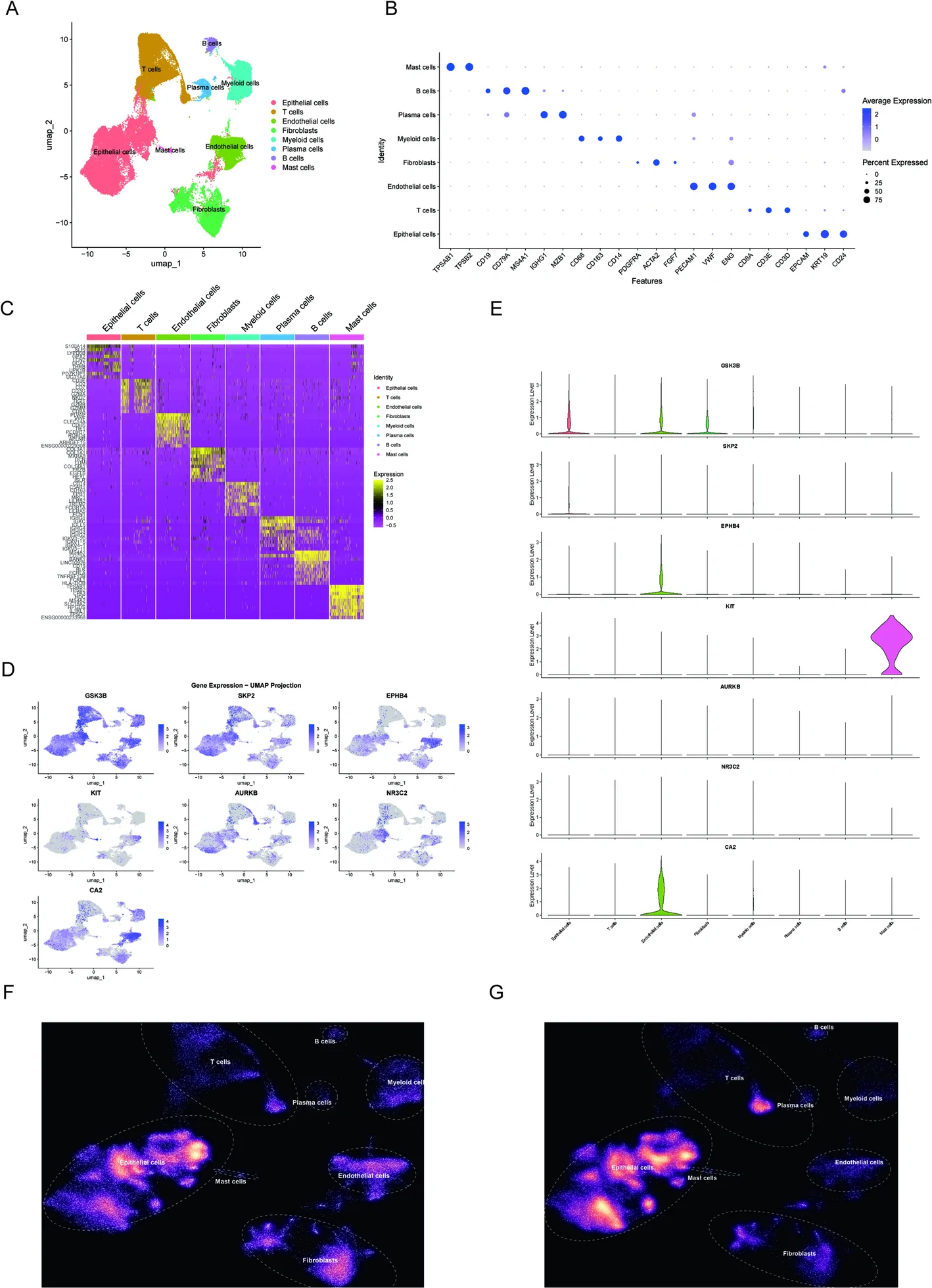
Single-cell transcriptomic analysis of DEBRGs in bladder cancer. **A** UMAP visualization of cells classified into eight major populations, including mast cells, B cells, epithelial cells, T cells, endothelial cells, fibroblasts, myeloid cells, and plasma cells. **B** Bubble plot depicting marker gene expression for distinct cell subpopulations; bubble size represents the proportion of cells expressing the gene, and color intensity indicates average expression level. **C** Heatmap displaying the top 10 differentially expressed genes within each cell population. **D**, **E** UMAP and violin plots illustrating the expression of selected genes across different cell subgroups. **F**, **G** The starburst diagram illustrates the expression patterns of GSK3B and SKP2 in different cell subgroups

Based on UMAP clustering, cells were classified into 8 major populations, including mast cells, B cells, epithelial cells, T cells, endothelial cells, fibroblasts, myeloid cells, and plasma cells (Fig. 6A). Dot plot analysis verified the cell type–specific expression of typical marker genes across these clusters, and heatmap was generated to display the top 10 differentially expressed genes for each cell population (Fig. 6B, C). To further characterize the cellular distribution of differentially expressed biomarker-related genes (DEBRGs), their expression patterns were systematically evaluated across individual cell types. The expression of DEBRGs was highly heterogeneous across cell populations, with both average expression levels and the fraction of positive cells varying considerably, reflecting strong cell type specificity (Fig. 6D, E). Notably, GSK3B was predominantly enriched in epithelial cells, endothelial cells, and fibroblasts, whereas SKP2 showed higher expression levels in epithelial cells and plasma cells. This distribution aligns with their established molecular functions in cell cycle regulation and kinase-mediated signal transduction (Fig. 6F, G).

### Molecular docking validation of BaP with the hub genes

**Fig. 7:**
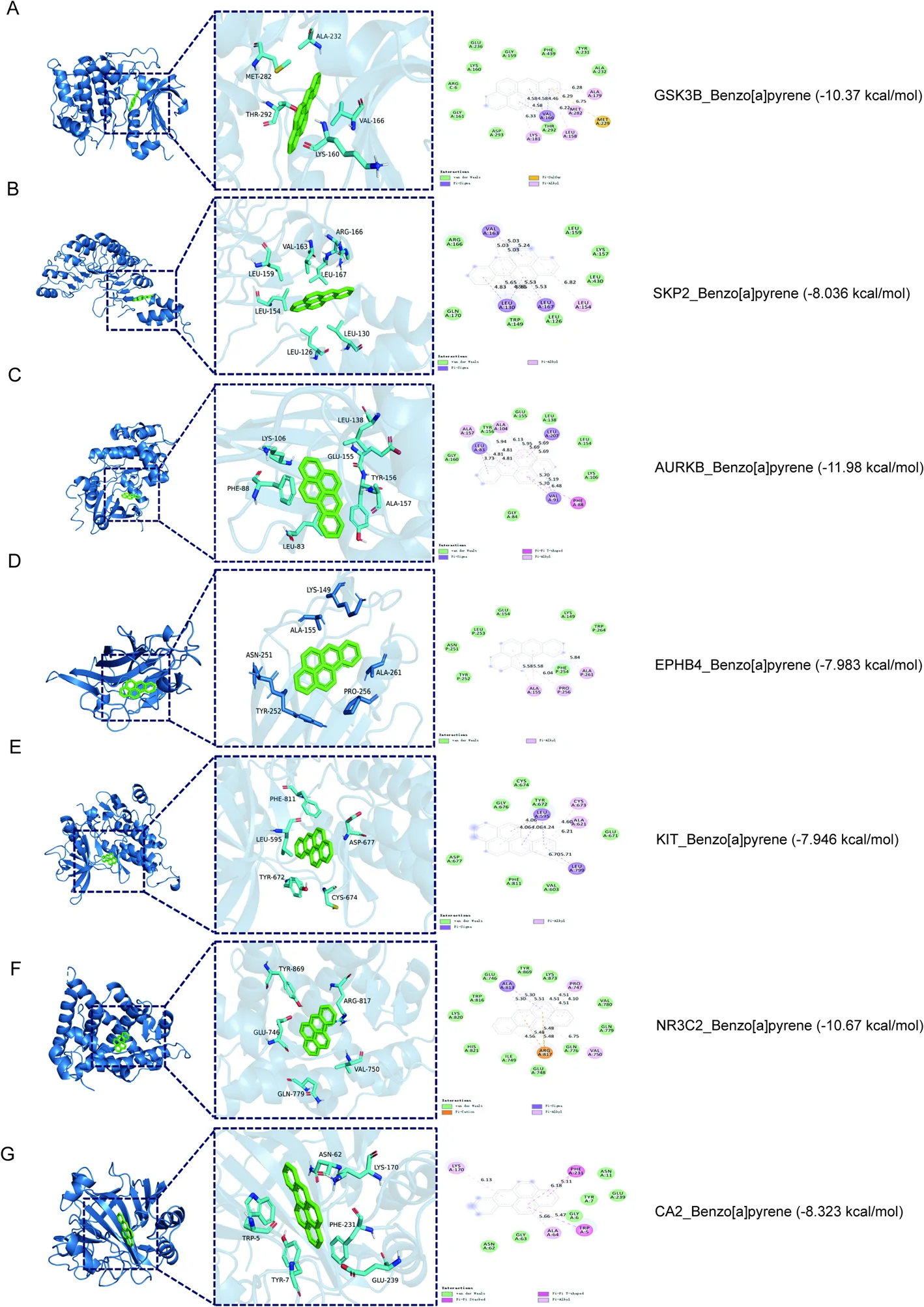
Molecular docking validation of BaP-core gene interactions. **A** Docking results for GSK3B bound to BaP. **B** Docking results for SKP2 bound to BaP. **C** Docking results for AURKB bound to BaP. **D** Docking results for EPHB4 bound to BaP. **E** Docking results for KIT bound to BaP. **F** Docking results for NR3C2 bound to BaP. **G** Docking results for CA2 bound to BaP

In order to investigate the possible interactions between BaP and potential proteins, we carried out molecular docking to determine binding free energies of BaP with DEBRGs and thus measure their binding affinities. The lower the binding free energy, the better and more stable the ligand-protein interactions are. It was found that there were significant variations in binding free energies between the targets, which varied between − 7.946 kcal/mol and − 11.98 kcal/mol. Visualizing the docking conformations revealed that all BaP-protein complexes took stable binding poses (Fig. 7A and G). These results suggest potential structural interactions between BaP and target proteins of DEBRGs identified using machine learning.

### Molecular dynamics simulations

**Fig. 8:**
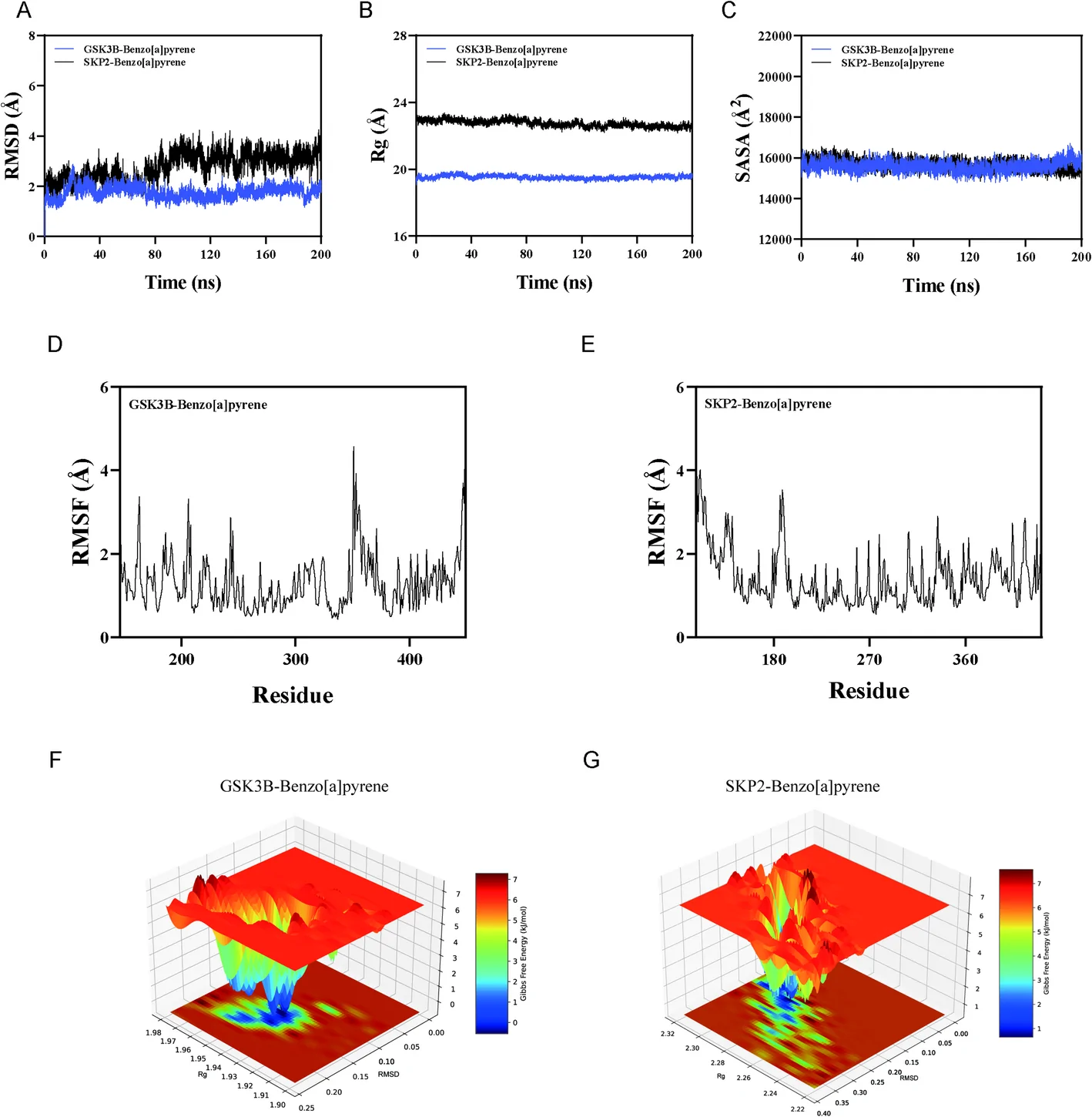
Molecular dynamics simulation of the protein-ligand complex. **A** RMSD value for protein-ligand complex over time. X-axis: simulation time (ns); Y-axis: RMSD (Å). **B** Rg value for protein-ligand complex over time. X-axis: simulation time (ns); Y-axis: Rg (Å). **C** SASA value for protein-ligand complex over time. X-axis: simulation time (ns); Y-axis: SASA (nm²). **D**, **E** RMSF value for protein-ligand complex over time. X-axis: residue number; Y-axis: RMSF (Å). **F**, **G** Landscape topography of binding free energy. X- and Y-axes: collective variables; Z-axis: free energy (kcal/mol)

To further verify the affinity between BaP and key proteins (GSK3B and SKP2), molecular dynamics simulations were carried out. The RMSD analysis showed that the GSK3B and SKP2-BaP complexes reached equilibrium at approximately 50 ns and 80 ns, respectively, with RMSD values stabilizing around 1.8 Å and 3.0 Å, indicating stable ligand-protein binding (Fig. 8A). During the simulation process, Rg and SASA were relatively stable (Fig. 8B, C), which means that the overall protein structure was not greatly affected by ligand binding. The analysis of RMSF showed that the flexibility of amino acid residues is low (mostly below 3 Å), indicating strong structural stability (Fig. 8D, E). In addition, the free energy diagram (FEL) based on RMSD and Rg reveals the favorable configuration of energy (Fig. 8F, G). Furthermore, MM-PBSA binding free energy calculations yielded consistently negative values for both complexes and highlighted the amino acid residues with important contributions to BaP binding, further corroborating the stable interactions observed in the simulations (Figure S2 A-C). These results indicate that BaP forms a stable complex with SKP2 and GSK3B, supporting their potential as BLCA-related targets.

## Discussion

BaP is a chemical compound commonly found in cigarette smoke, diesel exhaust dust, and cooked food. Being a very lipophilic compound, its metabolism is primarily performed by the liver via the CYP1 family. Some highly active metabolites can form adducts with DNA, showing significant mutagenic characteristics. According to massive experimentation in animals and epidemiological studies, the International Agency for Research on Cancer (IARC) categorized this molecule as a Class 1 carcinogen, which demonstrated its ability to be genetically toxic and carcinogenic in humans [14].

Exposure to BaP has the capacity to decrease the viability of the cell, cause DNA damage and lipid peroxidation, initiate numerous signaling pathways resulting in the up-regulation of p53 and ultimately results in blocking of cell cycle and death of the cell [15, 16]. There are a number of modalities of cell death caused by BaP that include apoptosis, necrosis, and ferroptosis [17–19]. The mitochondrial process of cell death is frequently recorded, entailing the activation of effector caspases 3 and 7, which may occur with or without the release of cytochrome c [20, 21]. Though these cytotoxic effects are widely presumed to have the positive impact of removing the severely damaged cells, growing evidence suggests that the killing of cells by agents that are genetically toxic carcinogens may also contribute to tumor propagation during chronic exposure conditions. Genetically toxic carcinogen induced cell death can relay mitotic signals to surrounding cells that undergo less damage to their DNA, thereby raising the likelihood of cell mutation. The cell death caused by carcinogens can eventually facilitate the growth of genetically and epigenetically altered cells in a competitive manner [16, 22]. This compensatory proliferation and a selective pressure potentially could permit the existence and proliferation of transformed cell groups, thereby favouring the early tumor development process. Our comprehensive analysis shows that BaP may exert its carcinogenic effect through multi-level molecular regulation. Besides established genetic toxicity, the BaP also appears to influence the required cell pathways which regulate proliferation, apoptosis and genome stability. Specifically, the disruption of mitosis mechanism and cell cycle regulatory factors is a critical contributing mechanism for BaP to promote the malignant transformation of bladder epithelial cells. This highlights the importance of considering direct DNA damage and broader regulatory network disturbances when assessing the impact of environmental carcinogens.

The recurrence rate of BLCA and the poor prognosis at the late stage are considered a significant health problem globally. The precise mechanism in which this environmental carcinogen causes tumor formation at the molecular level is yet to be completely understood. The study of these mechanisms plays a paramount role in understanding not only the causes of the disease, but also possible biomarkers and targets of therapy. While the primary carcinogenicity of BaP is mediated through metabolic activation and BPDE-DNA adduct formation, molecular docking and dynamics simulations were performed to explore potential associations with key proteins. These analyses are intended as hypothesis-generating tools to prioritize proteins for further study, rather than evidence of direct protein inhibition by BaP. Among the potential targets of BaP, GSK3B and SKP2 are the core mediators through which BaP may influence BLCA. In bladder cancer, GSK3B plays a dual role: on the one hand, it inhibits tumor growth by regulating the degradation of β-catenin; on the other hand, it can activate the NF-κB-mediated carcinogenic process [23, 24]. SKP2 is a core component of the ubiquitin-proteasome system, which can regulate the growth and drug resistance of cancer cells by modifying multiple proteins with ubiquitin, as well as controlling the cell cycle, signal transduction, and DNA repair [25, 26].

Combined interference with GSK3B and SKP2 during BaP exposure could potentially disrupt cell cycle regulation, mitosis fidelity, and DNA damage response, thereby generating vulnerabilities in regulatory networks that favor the selective survival and expansion of transformed bladder epithelial cells. Our analysis indicates that GSK3B and SKP2 may act synergistically in regulating the cell cycle and maintaining DNA stability. Specifically, GSK3B modulates G1/S transition and mitotic signaling through phosphorylation of key cell cycle proteins and transcription factors, such as Cyclin D and β-catenin, while also participating in the DNA damage response. SKP2, as a component of the SCF E3 ubiquitin ligase complex, promotes cell cycle progression by targeting cyclin-dependent inhibitors for ubiquitin-mediated degradation. Under BaP-induced stress, their coordinated activity may regulate cell cycle checkpoints, mitotic progression, and DNA repair, thereby creating conditions that facilitate the survival and proliferation of abnormal bladder epithelial cells. In parallel, BaP itself may globally perturb signaling networks associated with chromatin organization, transcriptional regulation, and kinase-mediated pathways, further destabilizing intracellular homeostasis and promoting abnormal proliferation. Collectively, these findings provide a mechanistic basis for the link between chronic BaP exposure and bladder carcinogenesis, highlighting that environmental carcinogens exert their effects not only through direct genotoxicity but also via disruption of regulatory networks.

Despite the insights gained from integrative computational analyses, this study has several limitations. The datasets utilized were derived from publicly available bulk and single-cell transcriptomic data rather than experimental models directly exposed to BaP, and thus the identified genes may reflect potential molecular associations rather than direct BaP-induced transcriptional changes. Additionally, BaP target genes were predicted using multiple platforms to improve coverage and reduce database-specific bias, although this approach may also increase the probability of false-positive predictions. Further experimental validation is required to confirm these predicted interactions, for instance, to elucidate the precise function of BaP target genes in bladder epithelial transformation. Moreover, longitudinal studies exploring the interactive effects of environmental exposure, genetic variation, and lifestyle are pertinent to gain a comprehensive understanding of the risk of BaP-related BLCA.

Overall, our study provides an in-depth perspective on the potential mechanisms by which BaP may promote bladder cancer development. Multiple analyses suggest that BaP exposure could perturb cell cycle regulation, mitotic progression, and key signaling pathways in bladder cancer cells, with GSK3B and SKP2 emerging as central nodes in these regulatory networks. These findings provide a framework for understanding the potential environmental carcinogenic role of BaP in bladder cancer and identify potential biomarkers as well as possible avenues for the development of targeted therapeutic strategies for BaP-induced BLCA.

## Conclusion

In summary, BaP, as an environmental toxicant, may promote bladder cancer development by perturbing key regulatory pathways that govern cell cycle progression and mitosis. Through our integrative analyses, GSK3B and SKP2 were identified as central mediators in BaP-induced bladder carcinogenesis. These findings provide mechanistic insights into the potential link between environmental BaP exposure and bladder cancer and offer a framework for understanding its environmental carcinogenic effects.

## Electronic Supplementary Material

Below is the link to the electronic supplementary material.

Supplementary Material 1.

## Data Availability

All data used in this study are publicly available. Bulk and single-cell transcriptomic datasets were obtained from the NCBI Gene Expression Omnibus (GEO; [https://www.ncbi.nlm.nih.gov/geo/](https:/www.ncbi.nlm.nih.gov/geo) ), including GSE13507, GSE31684, GSE32894, GSE7476, GSE37815, GSE65635, and GSE222315. Chemical information for benzo[a]pyrene was retrieved from PubChem (CID: 2336; [https://pubchem.ncbi.nlm.nih.gov/compound/2336](https:/pubchem.ncbi.nlm.nih.gov/compound/2336) ). Toxicity predictions were generated using ADMETlab 3.0 ( [https://admetlab3.scbdd.com/](https:/admetlab3.scbdd.com) ) and ProTox-III (https://tox.charite.de/protox\_III/). Target prediction analyses were performed using ChEMBL ( [https://www.ebi.ac.uk/chembl/](https:/www.ebi.ac.uk/chembl) ), SwissTargetPrediction ( [https://www.swisstargetprediction.ch/](https:/www.swisstargetprediction.ch) ), and PharmMapper (http://www.lilab-ecust.cn/pharmmapper/). Functional enrichment analyses were conducted using the clusterProfiler R package based on the Gene Ontology (GO; [https://geneontology.org/](https:/geneontology.org) ) and Kyoto Encyclopedia of Genes and Genomes (KEGG; [https://www.kegg.jp/](https:/www.kegg.jp) ) databases.
