# Supplementary material for: Integrating machine learning and structural analysis to decipher benzo[a]pyrene-induced bladder cancer networks
Source: Discov Oncol. 2026 May 21;17:1229. doi: 10.1007/s12672-026-05207-6 (PMC13500443; doi:10.1007/s12672-026-05207-6)
Supplement: Supplementary file 1 [file 12672_2026_5207_MOESM1_ESM.docx]

**Supplementary Figure.1. Performance validation of the predictive model.**

(A) ROC curve showing the discriminatory ability of the predictive model. (B) Calibration curve of the predictive model with apparent, bias-corrected and ideal curves. (C) Decision curve analysis (DCA) curve of the predictive model.

**Supplementary Figure.2. MM-PBSA binding free energy analysis of the GSK3B and SKP2-BaP complex.**

(A) Per-residue decomposition of the binding free energy. Negative values indicate favorable interactions. (B) Binding free energy components of GSK3B-BaP complex. (C) Binding free energy components of SKP2-BaP complex.
